# Lynch Syndrome Identification in Saudi Cohort of Endometrial Cancer Patients Screened by Universal Approach

**DOI:** 10.3390/ijms232012299

**Published:** 2022-10-14

**Authors:** Rong Bu, Abdul K. Siraj, Sandeep Kumar Parvathareddy, Kaleem Iqbal, Saud Azam, Zeeshan Qadri, Maha Al-Rasheed, Wael Haqawi, Mark Diaz, Khadija Alobaisi, Padmanaban Annaiyappanaidu, Nabil Siraj, Hamed AlHusaini, Osama Alomar, Ismail A. Al-Badawi, Fouad Al-Dayel, Khawla S. Al-Kuraya

**Affiliations:** 1 Human Cancer Genomic Research, Research Center, King Faisal Specialist Hospital and Research Center, Riyadh 11211, Saudi Arabia; 2 Department of Medical Oncology, King Faisal Specialist Hospital and Research Center, Riyadh 11211, Saudi Arabia; 3 Department of Obstetrics-Gynecology, King Faisal Specialist Hospital and Research Center, Riyadh 11211, Saudi Arabia; 4 Department of Pathology, King Faisal Specialist Hospital and Research Centre, Riyadh 11211, Saudi Arabia

**Keywords:** endometrial cancer, Lynch syndrome, mismatch repair-deficient, Lynch-like syndrome, universal screening, germline mutations, somatic mutations

## Abstract

Lynch syndrome (LS) is the most common cause of inherited endometrial cancer (EC). The prevalence and molecular characteristic of LS in Middle Eastern women with EC have been underexplored. To evaluate the frequency of LS in a cohort of EC patients from Saudi Arabia, a total of 436 EC cases were screened utilizing immunohistochemistry (IHC), *MLH1* promoter methylation analysis and next-generation sequencing technology. A total of 53 of 436 (12.2%) ECs were classified as DNA mismatch repair-deficient (dMMR). *MLH1* promoter hypermethylation was detected in 30 ECs (6.9%). Three ECs (0.7%) were found to be LS harboring germline pathogenic variants (PVs)/likely pathogenic variants (LPVs): two in the *MSH2* gene and one in the *MSH6* gene. Three ECs (0.7%) were Lynch-like syndrome (LLS) carrying double somatic *MSH2* PVs/LPVs. Seven cases were found to have variants of uncertain significance in cancer-related genes other than MMR genes. Our results indicate that LS prevalence is low among Saudi EC patients and LLS is as common as LS in this ethnicity. Our findings could help in better understanding of the prevalence and mutational spectrum of this syndrome in Saudi Arabia, which may help in defining best strategies for LS identification, prevention and genetic counseling for EC patients.

## 1. Introduction

Endometrial carcinoma (EC) is one of the leading causes of morbidity and mortality worldwide and its incidence is increasing each year [1,2]. In Saudi Arabia, EC is the fourth most common malignancy among women, accounting for 6.1% of all cancers in females (Cancer Incidence Report Saudi Arabia 2015). Approximately 5% of all EC cases are due to inherited cancer syndromes, with Lynch syndrome (LS) being the most commonly associated [3].

The diagnosis of LS in endometrial cancer is of great importance for counseling and cancer surveillance of patients and their families. LS is a hereditary, cancer-prone syndrome caused by germline pathogenic variants (PVs) or likely pathogenic variants (LPVs) in the DNA-mismatch repair (MMR) pathway genes (*MLH1*, *MSH2*, *MSH6* and *PMS2*) or the epithelial cell adhesion molecule (*EPCAM*) gene [4,5,6,7,8]. MMR deficiency (dMMR) leads to the accumulation of mismatches, insertions and deletions in short tandem repeats, causing microsatellite instability (MSI). Consequently, LS-associated tumors commonly exhibit MMR deficiency (dMMR), as reflected by high-level microsatellite instability (MSI-H) or loss of MMR protein expression, which are the main features of these tumors [9,10], whereas few cases are caused by biallelic somatic MMR gene inactivation and are termed “Lynch-like syndrome” (LLS) [11]. Despite advances in sequencing and screening approaches, there is a considerable percentage of MMR-deficient tumors that remains unexplained. Therefore, identifying EC patients with dMMR is of great importance to implement better surveillance strategies and could help in offering the required genetic counseling and testing for the family members of the affected individual.

In Saudi Arabia, screening for LS in EC depends only on clinical criteria. Therefore, the prevalence of LS and LLS among Saudi EC patients is not fully explored. To elucidate this, we sought to determine the frequency of LS and LLS in a Saudi population by conducting a retrospective study on 436 EC patients who have undergone surgical treatment in a single institution, as a step towards understanding the prevalence, molecular epidemiology and clinicopathological features of LS and LLS in EC in the Saudi population.

## 2. Results

### 2.1. Patient Characteristics

Median age of the study population was 59.3 years (range 25.6–91.6 years). A majority of tumors were type I (endometrioid adenocarcinoma) histology, accounting for 87.8% (383/436) of ECs, with an almost equal distribution among well-, moderately and poorly differentiated tumors. A majority of the cases were stage I tumors (64.4%; 281/436). Lymph-node metastasis was noted in 6.9% (30/436) of ECs, whereas distant metastasis was present in 5.3% (23/436) (Table 1).

### 2.2. Tumor Screening by IHC

The entire cohort was screened by IHC to detect dMMR. A total of 53 of 436 (12.2%) cases were identified to have loss of one or more protein expression of MMR genes. Most dMMR cases had loss of MLH1 protein expression (36/53, 67.9%), accounting for 8.3% of total cases, while 32 of 53 (60.4%) cases showed isolated loss of MLH1 and only four (7.5%) cases were negative for expression of MLH1 and PMS2. Isolated loss of PMS2 protein expression was seen in two of 53 (3.8%) cases. Loss of both MSH2 and MSH6 was detected in three (5.7%) cases. Isolated loss of MSH2 protein was observed in seven (13.2%) cases while isolated MSH6 loss was detected in five (9.4%) cases.

### 2.3. MLH1 Promoter Hypermethylation Analysis

*MLH1* promoter methylation was analyzed in all 36 cases with loss of MLH1 protein by IHC, and a majority of them (30/36, 83.3%) were hypermethylated, accounting for 56.6% of all dMMR cases. Endometrial tumors with *MLH1* promoter hypermethylation are considered sporadic; therefore, we excluded these from the germline variant analysis.

### 2.4. LS and LLS Analysis by Next-Generation Sequencing

Capture sequencing analysis was performed to identify germline variants in MMR genes among 23 cases without *MLH1* promoter hypermethylation. Germline PVs/LPVs were identified in three cases (0.7% out of 436 cases) including two cases carrying *MSH2* PVs (p.Q288X and p.Q409Rfs) and one case with LPV of *MSH6* (p.R772W). All three variants were reported by ACMG or ClinVar. We did not detect any germline PVs in *MLH1* or *PMS2* genes (Table 2). There were no variants of uncertain significance (VUS) in MMR genes detected in any of the sequenced cases.

Whole-exome sequencing analysis was carried out to identify the LLS cases and germline variants in cancer-related genes other than MMR genes among 20 cases. There were three cases identified to carry double somatic PVs/LPVs in the *MSH2* gene only. One case carried double somatic PVs of p.E530X and p.E580X. This case also harbored one somatic VUS (c.2156T > G:p.I719S) in the *MLH1* gene. A second case harbored double *MSH2* somatic PVs of p.S676X and p.E580X. Third case was identified to have double somatic p.C199R (LPV) and p.S77fs (PV) (Table 3). One single somatic variant of *MLH1* c.1989G > A:p.E633E was identified in one case. This variant falls at the last nucleotide of exon 17 of the *MLH1* coding sequence and classified as LPV by ClinVar. However, due to a lack of functional studies to support a damaging effect of this variant on *MLH1*, ACMG classifies this variant as VUS.

In addition, seven cases were found to carry one or more VUS in cancer-related genes: *ATM*, *BLM*, *CDK4*, *FANCF*, *FANCM*, *PMS1*, *RAD51C*, *RET* and *RINT1* (Table 4). However, there were no PVs or likely PVs detected in these cancer-related genes among the remaining 20 cases.

### 2.5. Copy-Number Variation (CNV) Analysis

CNV analysis was performed on germline whole-exome sequencing data and capture sequencing data to detect the copy-number deletion in *MLH1*, *MSH2*, *MSH6*, *PMS2* and *EPCAM* among 20 cases without *MLH1* promoter hypermethylation and germline PVs/LPVs. However, no copy-number deletions were identified in these genes among any of the analyzed cases.

### 2.6. Clinicopathological Characteristics of LS and LLS

Median age of the patients with LS in our cohort was 49 years (range 37–60 years), which is not significantly lower than the median age of sporadic ECs (59.0 years; *p* = 0.1264). All three patients with confirmed LS had stage I endometrioid adenocarcinoma: 66.7% (2/3) of these patients had moderately differentiated tumors, whereas 33% had poorly differentiated (1/3) tumors (Table 2). None of the three patients had either a family history or personal history of other cancers.

LLS was detected in three cases, with a median age of 55 years (range 49–78 years), which is not significantly lower than the median age of sporadic ECs (59.0 years; *p* = 0.9520); 33.3% (1/3) presented with stage I endometrioid adenocarcinoma and 66.7% (2/3) with stage III endometrioid adenocarcinoma. Of the three cases, one was well differentiated and two were poorly differentiated tumors (Table 3). None of the three LLS patients had either a family history or personal history of other cancers.

## 3. Discussion

This study confirms the prevalence of LS and LLS among a Saudi population-based EC cohort undergoing universal screening. We successfully screened 436 unrelated EC patients by IHC, of which 53 were dMMR tumors (12.2%). Thirty tumors (56.6%) were found to be sporadic due to *MLH1* promoter hypermethylation and the remaining 23 cases without *MLH1* hypermethylation were selected for germline PV/LPV analysis. We found germline MMR PV/LPV in three cases (0.7%) of the total cohort. Germline PVs in the *MSH2* gene was seen in two EC cases (66.7%), while one (33.3%) was *MSH6* LPV. The spectrum of PVs/LPVs in our cohort was different from those previously reported, in which PVs/LPVs in *MSH6* were most commonly detected in EC [12,13]. The prevalence of LS in this cohort was 0.7%, which is lower than what previous studies have shown. Previous reports have detected LS among EC patients of between 2% and 5.9% [12,13,14]. The lower frequency of LS and the different mutational spectrum among EC in this cohort could be partially explained by the difference in ethnicity and cohort size. However, further studies on the incidence of LS among EC patients of Middle Eastern ethnicity are needed. Furthermore, this study showed that double somatic MMR PVs/LPVs are as common as LS among EC patients (0.7%), This is important for planning genetic testing strategies for EC with dMMR tumors in this ethnicity.

Historically, Amsterdam criteria and Bethesda guidelines have been widely used to screen for LS, since personal and family histories of cancer are usually stronger in LS families. However, none of the LS and LLS cases in our cohort fulfilled these criteria. In addition, it has been reported that patients with LLS develop cancer at younger ages, similar to LS [15]. In our cohort, neither LS nor LLS presented at a significantly younger age compared to sporadic EC patients. These findings might have significant impact on practice guidelines for LS and LLS in Middle Eastern populations and suggest that EC patients need to be screened for LS and LLS irrespective of age at diagnosis and family history.

The typical approach for dMMR EC begins with germline MMR testing. However, 92.5% of the dMMR cases in this cohort did not have germline PVs or LPVs. Follow-up with next-generation sequencing (NGS) analysis is the only way to identify double somatic MMR PVs/LPVs. The comparable frequency of MMR germline PV/LPV and double somatic PVs/LPVs in our cohort indicates that NGS sequencing of paired normal and tumor tissue samples could be an appropriate follow-up approach for Saudi patients with dMMR tumors. Furthermore, exome sequencing helped us to identify seven patients carrying VUS in cancer-related genes other than MMR genes out of 16 patients who had neither LS nor LLS. None of these patients harboring VUS had a family history of cancer. The functional and clinical impact of these VUSs is not fully explored. Whether these VUSs in these genes play a role in the etiology of EC with dMMR needs to be further illustrated.

In our study, none of the dMMR EC patients was found to harbor copy-number deletion in MMR and *EPCAM* genes. This result is in concordance with a previous report that CNV in LS is rare and no germline copy-number deletions in MMR genes were observed among LS patients [16]. Therefore, this indicates that copy-number deletion in MMR genes did not contribute to EC in our population. In this study, CNV analysis was performed on the DNA extracted from normal FFPE tissue, and artifacts might have occurred due to DNA degradation and deamination of cytosine bases caused by long-term storage, fixation and embedding conditions. Therefore, this result should be interpreted with caution.

This study has some limitations, since it was conducted at a single institute and might not reflect LS among EC in general population. Secondly, although using universal screening helped in identifying cases of LLS, the recommendation of its implementation for LS and LLS among the Middle Eastern EC population is challenging when resources for hereditary cancer diagnosis and genetic counseling are still unsatisfactory.

In conclusion, this is the first study on a Saudi EC cohort of screened for LS using universal screening, which revealed an LS prevalence of 0.9% and LLS of 0.7%. LS identification in this population is important for clinicians to better estimate and potentially tailor treatment and preventive strategies. The lack of difference in age at diagnosis between LS/LLS and sporadic EC in this ethnicity indicates that screening EC without an upper age limit to identify LS may benefit counseling and facilitate prevention strategies for EC patients and their families.

## 4. Materials and Methods

### 4.1. Patients and Samples

**Figure 1:**
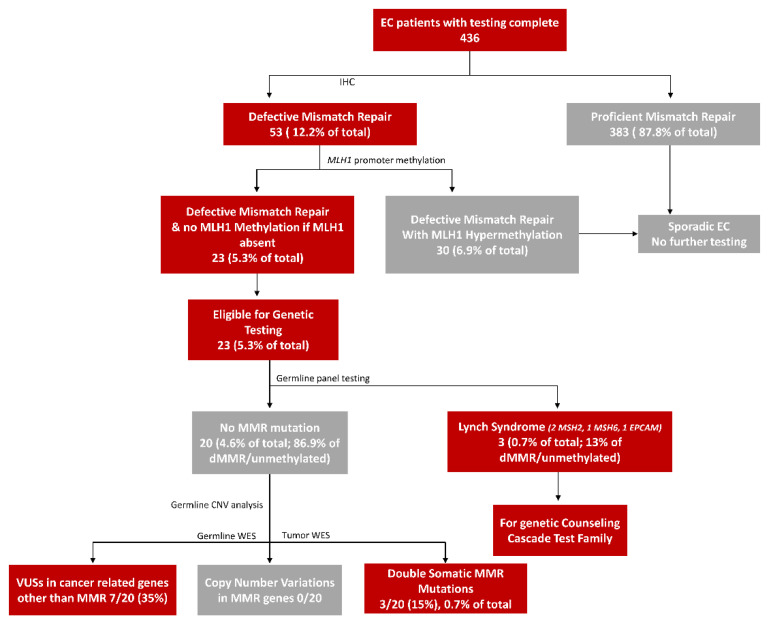
Endometrial cancer study schema.

A total of 436 unselected EC cases diagnosed between 1990 and 2016 from King Faisal Specialist Hospital and Research Centre, Riyadh, Saudi Arabia were included in the study. Detailed clinicopathological data, including follow-up data, were noted from case records and are summarized in Table 5. All tissue samples were obtained from patients with approval from the Institutional Review Board (IRB) of the hospital. Waiver of consent was obtained for all archived paraffin tissue blocks, including normal tissue blocks used as controls, from the IRB of King Faisal Specialist Hospital and Research Centre under project RAC 2180 001. The overall testing schema utilized in this study is shown in Figure 1.

### 4.2. Tissue Microarray (TMA) Construction and Immunohistochemistry Assessment

All samples were analyzed in a tissue microarray (TMA) format. TMA construction was performed as described earlier [17]. Briefly, tissue cylinders with a diameter of 0.6 mm were punched from representative tumor regions of each donor-tissue block and brought into recipient paraffin blocks using a modified semiautomatic robotic precision instrument (Beecher Instruments, Woodland, WI). Two cores of EC were arrayed from each case.

Immunohistochemical staining of MMR proteins (MLH1, MSH2, MSH6 and PMS2) were performed manually. The primary antibodies used and their dilutions are shown in Table 2. Tumors were classified as dMMR if any of the four proteins showed loss of staining in cancer with concurrent positive staining in the nuclei of normal epithelial cells. Otherwise, they were classified as proficient MMR (pMMR).

IHC scoring was done by two pathologists blinded to the clinicopathological characteristics. Discordant scores were reviewed together to achieve agreement.

### 4.3. DNA Extraction

DNA samples were extracted from formalin-fixed and paraffin-embedded (FFPE) EC tumor and normal tissue (as far as possible from the endometrial carcinomas, such as fallopian tubes, cervix, ovaries, or uninvolved lymph nodes) utilizing a Gentra DNA Isolation Kit (Gentra, Minneapolis, MN, USA) according to the manufacturer’s protocols as elaborated in a previous study [18].

### 4.4. Bisulfite Modification of DNA and Real-Time PCR (MethyLight) for Quantitative MLH1 Promoter Methylation Analysis

Bisulfite modification of DNA samples followed by real-time PCR for the determination of *MLH1* methylation status was performed as described previously [19]. We used an ABI 7900HT Fast Real Time Analysis System (Applied Biosystems) for quantitative real-time PCR. A set of forward and reverse primers and probes were used to amplify promoter regions of *MLH1* and *COL2A1* (the collagen 2A1 gene) to normalize for the amount of input bisulfite-converted DNA. Primers and probes were previously validated and published [20,21]. The percentage of methylated reference (PMR) as described in [22] was calculated by dividing the *MLH1*:*COL2A1* ratio of a sample by the *MLH1*:*COL2A1* ratio of CpG methyltransferase-treated human genomic DNA (assuming it was fully methylated) and multiplying it by 100. A PMR cutoff of 4 was established to distinguish methylation positivity (PMR > 4) from methylation negativity (PMR ≤ 4). The PMR cutoff of 4 as previously described [23] was used for *MLH1*.

### 4.5. Capture Sequencing Analysis

Targeted capture sequencing was performed on 23 normal DNA samples using the Illumina platform with a custom-designed panel at a median depth of 2053x (range 1369–3026). All the quality metrics were applied as described previously [24]. ACMG and/or ClinVar were utilized for pathogenicity assessment of the variants identified.

### 4.6. Whole-Exome Sequencing

DNA samples extracted from normal and tumorous FFPE tissues were analyzed in 20 cases by whole-exome sequencing using Illumina Novaseq at median depth 157x (range 138–178) for tumor samples and 154 × (range 141–177) for paired normal tissue. Sequencing reads in Fastq format were mapped to the human genome version 19 using Burrows-Wheeler Aligner (BWA) [25]. PCR duplicate marking, local realignment and base-quality recalibration were performed with Picard tools (http://broadinstitute.github.io/picard/) and GATK [26].

Single-nucleotide variants (SNVs) and indels were called with MuTect [27] and VarScan2 (http://varscan.sourceforge.net), respectively. Annotation of somatic variants was performed using ANNOVAR [28]. The SNVs that passed the standard Mutect and VarScan2 filters were retained, and common SNPs with minor allele frequency (MAF) of > 0.001 in dbSNP, the NHLBI exome sequencing project, 1000 Genomes and our in-house exome database of around 800 normals were removed for further analysis. Somatic SNVs were manually checked using Integrated Genomics Viewer (IGV) to filter out the artifacts.

To predict germline, copy-number loss or gains, DELLY (v0.9.1), which is based on split-read and paired-end algorithms, was utilized with default parameters on targeted capture sequencing data [29]. Low-quality calls were filtered out by selecting the “PASS” filter.

### 4.7. Sanger Sequencing Analysis

Sanger sequencing technology was utilized to validate the variants identified by capture sequencing technology and whole-exome sequencing technology. Primer 3 online software was utilized to design the primers (available upon request). PCR and Sanger sequencing analyses were carried out as described previously [30]. Reference sequences were downloaded from the NCBI GenBank and sequencing results were analyzed using Mutation Surveyor V4.04 (Soft Genetics, LLC, State College, PA, USA).

### 4.8. Statistical Analysis

The median age of LS, LLS and sporadic EC patients was compared using Mann–Whitney U test (IBM SPSS Statistics, v.21).

## Figures and Tables

**Table 1:** Clinicopathological variables for the patient cohort (n = 436).

Clinicopathological Parameter	n (%)
**Age (years)**	
Median (range)	59.3 (25.6–91.6)
≤60	234 (53.7)
>60	202 (46.3)
**Histologic subtype**	
Type I	383 (87.8)
Type II	53 (12.2)
**Histological grade**	
Well differentiated	145 (33.3)
Moderately differentiated	148 (33.9)
Poorly differentiated	128 (29.4)
Unknown	15 (3.4)
**pT**	
T1	304 (69.7)
T2	54 (12.4)
T3	58 (13.3)
T4	19 (4.4)
Unknown	1 (0.2)
**pN**	
N0	406 (93.1)
N1-2	30 (6.9)
**pM**	
M0	412 (94.5)
M1	23 (5.3)
Unknown	1 (0.2)
**TNM Stage**	
I	281 (64.4)
II	47 (10.8)
III	70 (16.1)
IV	37 (8.5)
Unknown	1 (0.2)

**Table 2:** Clinicopathological characteristics of three Lynch syndrome cases in endometrial carcinoma.

S. No.	Age	Histologic Subtype	Grade	T	N	M	Stage	MSI Loss (IHC)	MMR Germline Mutation	HGVS	Pathogenicity
1	37	Endometrioid adenocarcinoma	Grade 2	T1	N0	M0	I	*MSH6*	*MSH6*	c.2314C > T:p.R772W	Likely Pathogenic
2	49	Endometrioid adenocarcinoma	Grade 3	T1	N0	M0	I	*MSH2*	*MSH2*	c.862C > T:p.Q288X	Pathogenic
3	60	Endometrioid adenocarcinoma	Grade 2	T1	N0	M0	I	*MSH2*	*MSH2*	c.1226_1227delAG:p.Q409Rfs	Pathogenic

**Table 3:** Clinicopathological details of the three Lynch-like syndrome cases.

S. No.	Age	Histologic Subtype	Grade	T	N	M	Stage	MSI Loss (IHC)	MMR Double Somatic Mutation	HGVS	Pathogenicity
1	49	Endometrioid adenocarcinoma	Grade 3	T2	N1	M0	III	*MSH2*	*MSH2*	c.1588G > T:p.E530X	Pathogenic
										c.1738G > T:p.E580X	Pathogenic
2	78	Endometrioid adenocarcinoma	Grade 3	T1	N0	M0	I	*MSH2*	*MSH2*	c.2027C > A:p.S676X	Pathogenic
										c.1738G > T:p.E580X	Pathogenic
3	55	Endometrioid adenocarcinoma	Grade1	T2	N0	M0	III	*MSH2*	*MSH2*	c.595T > C:p.C199R	Likely Pathogenic
										c.229_230del:p.S77fs	Pathogenic

**Table 4:** Clinicopathological details of cases carrying variants of uncertain significance in cancer-related genes other than MMR genes.

S. No.	Age	Histologic Subtype	Grade	T	N	M	Stage	HGVS
1	34	Endometrioid adenocarcinoma	Grade 1	T3	N0	M0	III	*RINT1* c.954G > C:p.R318S
								*CDK4* c.1091C > T:p.A114V
2	50	Mucinous carcinoma	Grade 1	T1	N0	M0	I	*PMS1* c.2167G > A:p.E723K
								*ATM* c.6101G > A:p.R2034Q
3	66	Endometrioid adenocarcinoma	Grade 3	T1	N0	M0	I	*PMS1* c.178G > A:p.G60S
								*RET* c.1785G > T:p.E595D
								*FANCF* c.860A > G:p.Y287C
4	56	Endometrioid adenocarcinoma	Grade 2	T2	N1	M0	III	*ATM* c.590G > A:p.G197E
								*RAD51C* c.431T > C:p.I144T
5	46	Endometrioid adenocarcinoma	Grade 3	T1	N1	M0	III	*RAD51C* c.431T > C:p.I144T
6	39	Endometrioid adenocarcinoma	Grade 3	T2	N0	M0	II	*FANCM* c.808C > T:p.R270C
7	59	Endometrioid adenocarcinoma	Grade 3	T2	N0	M0	II	*BLM* c.503C > T:p.S168L

**Table 5:** Antibodies used for the mismatch-repair immunohistochemistry assay.

Antibody	Clone	Source	Antigen Retrieval	Visualization System	Dilution
MSH2	FE11	Oncogene/CalBiochem	Dako retrieval solution (pH 9)	Dako EnVision+	1:100 overnight
MSH6	44	BD Transduction Laboratories	Dako retrieval solution (pH 9)	Dako EnVision+	1:100 overnight
MLH1	G168–15	BD Pharmingen	Dako retrieval solution (pH 9)	Dako EnVision+	1:50 overnight
PMS2	C-20	Santa Cruz Biotechnology	Dako retrieval solution (pH 9)	Dako EnVision+	1:100 overnight

## Data Availability

The data presented in this study are available in the form of tables in the manuscript.
